# Supporting Oral and Long-Acting HIV Preexposure Prophylaxis Decision-Making Among Pregnant Women (MyChoice Intervention): Protocol for 2 Pilot Randomized Controlled Trials

**DOI:** 10.2196/76442

**Published:** 2025-11-13

**Authors:** Friday Saidi, Symone Welch, Twambilile Phanga, Carlie Sulpizio, Lusubiro Paile, Doris Ngo’ma, Mercy Tsidya, Mary Chindebvu, Grace Nkhoma, Lovemore Nkhalamba, Ivy Kaliati, Nomthata Mkochi, Humphrey Chakala, Wiza Kumwenda, Agatha Bula, Picrina Winner, Tapiwa Munthali, Alinda Nyamaizi, Jessica Keys, Suzanne Maman, Lisa Pearce, Carol Golin, Benjamin H Chi, Lauren M Hill

**Affiliations:** 1 UNC Project Malawi Lilongwe Malawi; 2 School of Medicine University of North Carolina at Chapel Hill Chapel Hill, NC United States; 3 Gillings School of Global Public Health University of North Carolina at Chapel Hill Chapel Hill United States; 4 Department of Sociology University of North Carolina at Chapel Hill Chapel Hill United States

**Keywords:** pre-exposure prophylaxis, shared decision-making, pregnancy, prevention, choice, HIV

## Abstract

**Background:**

HIV preexposure prophylaxis (PrEP) has potential for preventing HIV during the perinatal period, but few strategies promote person-centered shared decision-making (SDM) about PrEP use in these contexts. The MyChoice study aims to evaluate the feasibility, acceptability, and appropriateness of an SDM approach to support pregnant and breastfeeding women in Lilongwe, Malawi, integrating PrEP into antenatal care to encourage consistent use.

**Objective:**

Two pilot studies will assess the feasibility, acceptability, and appropriateness of an SDM intervention to support pregnant and breastfeeding women in making informed HIV prevention choices. The intervention will also explore the perspectives of participants, their male partners, and implementers. Each study compares the MyChoice intervention to standard-of-care PrEP counseling, with different HIV prevention method choices available.

**Methods:**

We will conduct 2 pilot randomized trials to evaluate the MyChoice intervention, designed to support PrEP decision-making among pregnant women in Lilongwe. Both studies will compare the MyChoice intervention to standard-of-care PrEP counseling. Study 1 will include 100 participants who will be offered oral PrEP and condoms for 3 months. Study 2 will include 50 participants who will be offered oral PrEP or injectable long-acting cabotegravir and condoms for 2 months. The primary end points for MyChoice study 1 are intervention acceptability, intervention appropriateness, and intervention feasibility, whereas for MyChoice study 2 they are intervention acceptability and intervention appropriateness. The secondary end point for MyChoice study 1 is decisional conflict, and for MyChoice study 2, it is the feasibility of study procedures. Exploratory end points include PrEP uptake assessment and dried blood spot measurement of PrEP adherence for MyChoice study 1 and PrEP uptake assessment for MyChoice study 2.

**Results:**

Data collection started on July 30, 2024, for study 1 and on February 10, 2025, for study 2. A total of 100 participants were enrolled in MyChoice study 1, and 50 participants were enrolled in MyChoice study 2. Study follow-up has been completed. Data analysis is expected to be completed by November 2025.

**Conclusions:**

The MyChoice pilot studies will provide critical evidence on a novel intervention for PrEP decision-making during pregnancy and breastfeeding. If feasible and acceptable, these results will form the basis for larger efficacy trials to promote PrEP uptake during pregnancy.

**Trial Registration:**

ClinicalTrials.gov NCT06394323; https://clinicaltrials.gov/study/NCT06394323 and ClinicalTrials.gov NCT06397690; https://www.clinicaltrials.gov/study/NCT06397690

**International Registered Report Identifier (IRRID):**

DERR1-10.2196/76442

## Introduction

### Background

HIV preexposure prophylaxis (PrEP) is a proven yet underused tool to eliminate mother-to-child HIV transmission. With high adherence, daily oral PrEP with tenofovir-emtricitabine is highly efficacious in preventing HIV infection [1-3], including among expectant mothers, and it is safe to use during pregnancy and breastfeeding [4]. However, despite its promise, significant implementation challenges exist [5]. There is evidence suggesting that PrEP may be highly acceptable among PrEP-naive women without HIV, yet attrition and nonadherence are significant challenges for women using PrEP [6-10]. Women in PrEP trials have had consistently poor adherence despite adherence support [11]. Promoting user fit may be critical to achieve prevention-effective PrEP use among pregnant and breastfeeding women [12]. Recent advancements in biomedical HIV prevention have introduced the injectable PrEP option of long-acting cabotegravir (CAB-LA). Injectable formulations offer the benefits of a lower adherence burden and discreet use, particularly well suited for pregnant and breastfeeding women with complex health care needs [13,14]. However, use of injectable PrEP methods still requires user effort to return for repeat injections. Thus, engaging patients in the choice between different PrEP methods will still be critical to ensure patient engagement for persistence and adherence.

### Objectives

To ensure that medical decisions such as the choice between PrEP formulations and competing HIV prevention options align with a patient’s values and preferences, patient-centered counseling approaches such as shared decision-making (SDM) are recommended. SDM is a collaborative process in which patients and clinicians work together to make informed health care choices [12]. Few SDM or other patient-centered approaches for PrEP decision counseling exist; those developed to date have focused on male and nonpregnant US-based populations [15,16]. To address this gap, we developed the MyChoice intervention to support PrEP decision-making among pregnant women in the context of antenatal care in Malawi. MyChoice is a counselor-delivered SDM intervention facilitated by a paper-based patient decision aid. We developed this intervention through formative work to understand women’s values and preferences for PrEP decision-making in the context of pregnancy [17,18] and user testing. Through this process, we developed 2 versions of the intervention: one that only addresses oral PrEP as an option and another that addresses both daily oral PrEP and injectable CAB-LA as options. In parallel feasibility studies, we aim to evaluate the feasibility, acceptability, and appropriateness of each version of the MyChoice SDM intervention to support informed decisions about different PrEP modalities during pregnancy in Lilongwe, Malawi.

## Methods

### Study Context

The MyChoice intervention was developed with input from women receiving antenatal care at Bwaila District Hospital in Lilongwe, Malawi. We conducted extensive formative research with pregnant women, male partners, and clinicians from the study site to develop the intervention for these pilot studies. The findings of this research (regarding women’s values for PrEP decision-making, the implications of these values for feelings regarding PrEP use, and preferences for partner involvement [17]), as well as key guidelines (the Ottawa Decision Support Framework [19] and International Patient Decision Aid Standards Collaboration [12]) informed the development of the PrEP SDM intervention, known as MyChoice for HIV Prevention (MyChoice). The intervention was further refined ahead of these pilot studies through an iterative process of clinician and stakeholder feedback followed by feedback from 15 patient participants.

### Study Location

This study will take place at a government health facility, Bwaila District Hospital, a high-volume district facility run by the Malawi Ministry of Health. Approximately 1500 babies are delivered each month at Bwaila District Hospital, and approximately 500 women present each month for immunization visits for their babies at 9 months after delivery. Bwaila District Hospital has provided prevention of mother-to-child transmission services since April 2002 and has promoted male participation since 2007.

### Target Population

HIV-negative pregnant women are the primary population to be recruited for this study and will need to meet the following inclusion criteria: age of ≥18 years, a documented pregnancy confirmed via a urine pregnancy test or physical examination, and a documented HIV-negative status within the previous 3 months (Textbox 1). Participants should have factors indicating elevated risk of HIV acquisition per the Malawi national PrEP eligibility guidelines. These include a current or recent sexually transmitted infection within the previous 6 months (self-reported or clinically diagnosed), engagement in transactional condomless sex (including sex workers and clients), condomless sex with an HIV-infected partner not on antiretroviral therapy (ART), or with "Unknown viral load", condomless sex with a partner of unknown HIV status who has other high-risk partners, anal intercourse with a nonregular partner without a condom, or injecting drug use with needle sharing [20]. Participants must demonstrate a willingness to remain within the study site catchment area, comply with the visit schedule, and provide informed consent. Exclusion criteria include a positive HIV test at the time of screening, absence of HIV risk factors per Malawi national PrEP eligibility guidelines, and any risk of intimate partner violence or social harm from participating in the study as judged by study staff.

**Eligibility criteria.**

**Pregnant women inclusion criteria**

- Age ≥18 years
- Documented pregnancy via urine pregnancy test or physical examination
- Documented negative HIV status within the previous 3 months
- Identified factors for elevated risk of HIV acquisition per national preexposure prophylaxis (PrEP) eligibility guidelines
- Willingness to remain in the study site’s catchment area over the course of the study follow-up and to comply with the visit schedule
- Ability and willingness to provide informed consent

**Pregnant women exclusion criteria**

- Positive HIV test at the time of screening
- No identified HIV risk factors per national PrEP eligibility guidelines
- Risk of intimate partner violence or social harms as a result of participation per the judgment of the study personnel

**Male partner inclusion criteria (in-depth interviews only)**

- Referred by a study participant as her partner
- Age ≥18 years
- Ability and willingness to provide informed consent

**Study staff inclusion criteria (in-depth interviews only)**

- All study staff
- Ability and willingness to provide informed consent

### Study Design

We will evaluate each version of the intervention in 1 of 2 separate pilot feasibility studies. In study 1, a total of 100 pregnant women will be randomized 1:1 to receive either the SDM intervention addressing daily oral PrEP and alternative HIV prevention methods (condoms) or standard-of-care (SOC) counseling addressing the same prevention methods. In study 2, a total of 50 pregnant women will be randomized 1:1 to receive either the SDM intervention addressing daily oral PrEP, CAB-LA, and alternative HIV prevention methods (internal and external condoms) or SOC counseling addressing the same prevention methods. In both studies, we will evaluate the acceptability and appropriateness of the intervention. Pregnant women who express interest in either PrEP method will be referred to government PrEP services. Table 1 summarizes the study design. Study 1 was conceived before the CAB-LA rollout, with the sample size powered to detect an intervention effect on the secondary end point of decisional conflict. As CAB-LA became a viable option, we expanded the intervention to include it and secured funding to evaluate this expanded version. Study 2 focuses solely on evaluating intervention acceptability and appropriateness and feasibility of study procedures (no evaluation of intervention effect). Therefore, we selected a sample size appropriate for feasibility studies, aligning with similar research.

**Table 1:** Study design summary.

	Study 1	Study 2
Sample size	100 women	50 women
Intervention	MyChoice SDM^a^ counseling with choice of oral PrEP^b^ or condoms	MyChoice SDM counseling with choice of oral PrEP, CAB-LA^c^, or condoms
Follow-up duration	3 months	2 months
Primary end points	Intervention acceptability, appropriateness, and feasibility	Intervention acceptability and appropriateness
Secondary end points	Decisional conflict	Feasibility of study procedures
Exploratory end points	PrEP uptake assessment and dried blood spot assessment of PrEP adherence	PrEP uptake assessment

^a^SDM: shared decision-making.

^b^PrEP: preexposure prophylaxis.

^c^CAB-LA: long-acting cabotegravir.

### Study Intervention

The study intervention, MyChoice, is a counselor-delivered SDM tool for pregnant women considering PrEP (Table 2). The counseling sessions last approximately 45 minutes. Three counselors will deliver the intervention after undergoing training spanning 3 days and including a mix of content review and practical exercises to deliver counseling.

**Table 2:** Shared decision-making intervention content.

Counseling component			Content
Precounseling			Overview of counseling content Discussion of the participant’s desire to include their partner in counseling
**MyChoice counseling**			
	HIV information and risk assessment	HIV risk in pregnancy and breastfeeding Evaluation of HIV risk criteria according to Malawian national guidelines	
	HIV prevention options	Present oral PrEP^a^ and condoms Feature attributes and potential advantages and disadvantages of each	
	Value clarification	Identify and discuss participant-valued features of available options (attributes and personal and interpersonal implications of each method)	
	Structured deliberation	Assess and address unmet decision-making needs Collaboratively determine whether the participant is ready to decide and identify preferred methods	
	Postdecision counseling	Persistence or adherence counseling Disclosure coaching if desired	
	PrEP referral	If oral PrEP is selected, referral to SOC^b^ services or to an ongoing cohort study If CAB-LA^c^ is selected, referral to an ongoing cohort study	
Postcounseling			Adherence counseling for selected HIV prevention method Disclosure support Partner ART^d^ adherence counseling (if applicable)

^a^PrEP: preexposure prophylaxis.

^b^SOC: standard of care.

^c^CAB-LA: long-acting cabotegravir.

^d^ART: antiretroviral therapy.

Before the counseling visit, women will receive information about the content of MyChoice counseling and discuss whether they wish to have their partner or another person (eg, another family member) participate in counseling alongside them. MyChoice counseling may be conducted with any accompanying supporters depending on the participant’s preference. MyChoice counseling begins with a review of HIV risk in pregnancy and breastfeeding and includes discussion of the population-specific risk factors that may apply to the participant. After the participant understands her HIV risk, the counselor will present various HIV prevention options, including relevant PrEP methods and internal and external condoms, discussing their attributes, advantages, and disadvantages. Following the discussion of the HIV prevention options, a value clarification exercise will be implemented to help the participant identify which features of the prevention options offered matter most to her (product attributes and personal and interpersonal implications of each method). The counselor will then review the responses to the exercise and valued features for each method offered with the participant. The value clarification exercise will be used to understand and address any unmet participant decision support needs and will serve as the basis for structured deliberation to collaboratively identify the participant-preferred HIV prevention methods. The participant may defer or decline to make an HIV prevention method decision and may request a follow-up counseling visit, which will allow the participant to take more time to consider her preference or return with a partner or other supporter. When a decision is made, the counselor will provide postdecision counseling, including adherence counseling and disclosure counseling if desired.

Participants who choose PrEP will be asked whether they hope to disclose their decision to anyone. If yes, the participant will be offered additional coaching to support disclosure. Participants who have a partner living with HIV presently will receive brief counseling on the continued importance of ART adherence and mutual support for ART and PrEP adherence (if applicable). While counseling is intended to take place on 1 day, if the participant requests follow-up counseling (eg, wants more time to decide or wishes to come back with a partner for a future session), reasonable accommodations will be made for the MyChoice counselor to meet with the participant a second time. Women who choose PrEP during the intervention counseling session will be referred to government services to initiate PrEP (daily oral tenofovir disoproxil fumarate/emtricitabine) or to an ongoing observational cohort study that is evaluating the impact of PrEP on pregnancy, infant, and maternal health outcomes (CAB-LA or daily oral tenofovir disoproxil fumarate/emtricitabine) [21].

### SOC (Control Arm)

Participants randomized to the control arm will receive PrEP counseling delivered by a trained study nurse following the current SOC per Malawi national guidelines. SOC counseling includes the following elements: HIV risk assessment according to PrEP eligibility criteria, discussion of a combination prevention approach (PrEP and condoms), and risk reduction strategies that are not discussed in SDM. Participants will receive comprehensive education on both the advantages and limitations of PrEP, including guidance on managing potential side effects. Subsequently, the counselor will assess the woman’s eligibility, willingness, and readiness to start using PrEP. Women who choose oral PrEP during the SOC counseling session will be referred accordingly. No SDM counseling is provided in the SOC arm.

### Recruitment and Enrollment

We will recruit 100 eligible women for study 1 and 50 eligible women for study 2. Study staff will provide interested participants with study information and a referral to the study. Potential participants will be identified at any point in their pregnancy. All women attending the study site who meet the eligibility criteria will be invited to participate in the study. All male partners of invited female participants will be identified and invited to participate. Study staff will also be eligible for recruitment. Prospective pregnant women who choose to complete enrollment on the same day as screening or require additional time to return for the enrollment visit will be accommodated. Enrollment will be finalized upon completion of the informed consent process.

In addition to the primary sample, for qualitative data collection, we will enroll male partners and health care providers (including counselors) for in-depth interviews (IDIs) to assess their perception of feasibility and acceptability. The intended qualitative sample size is summarized in Table 3, although the final sample size will depend on theme saturation. Male partners are eligible for interviews if they are referred by a study participant as her partner, are aged ≥18 years, and provide informed consent. Study staff are also eligible to participate in interviews.

**Table 3:** Study sample size summary by participant type.

Type of participant	Study 1	Study 2
Pregnant women	100 (up to 30 complete IDIs^a^)	50 (up to 20 complete IDIs)
Male partners of pregnant PrEP^b^ users (IDIs only)	Up to 20	Up to 10
PrEP counselors or health care workers (IDIs only)	Up to 15	Up to 15

^a^IDI: in-depth interview.

^b^PrEP: preexposure prophylaxis.

### Randomization

In each study, pregnant women will be randomly assigned to either the intervention or SOC comparison arm. Health care providers and male partners will not be randomized but will participate only in the qualitative component. Randomization will be conducted using statistical software to generate a 1:1 allocation ratio. The assignments will be placed in opaque, sealed envelopes and sequentially numbered with participant identification numbers.

### Study Follow-Up

Participating pregnant women will receive their selected PrEP method (if applicable) through existing services or studies outside the scope of this study. Initiation of either PrEP method (ie, receipt of prescription) will be confirmed through clinic or pharmacy records, and information on reasons for noninitiation, if applicable, will also be collected. In study 1, we will conduct follow-up visits at months 1, 2, and 3 after enrollment (Table 4). Study 2 will have follow-up visits at months 1 and 2 after enrollment (Table 5). Study visits will be scheduled to align with antenatal care or pharmacy visits whenever possible to minimize the number of trips needed to the clinic.

**Table 4:** Schedule of evaluations for study 1.

				Screening		Enrollment		Month 1		Month 2		Month 3
**Eligibility assessment**												
	Pregnancy status			✓								
	HIV status			✓								
	Additional eligibility criteria			✓								
**Participant-reported assessments**												
	**Questionnaires**											
		Demographic information			✓							
		Intervention assessment			✓				✓			
		Decisional conflict			✓							
		Partner information			✓		✓		✓		✓	
		Social harms			✓		✓		✓		✓	
		Qualitative interviews					✓				✓	
**Oral PrEP^a^ adherence assessment**												
	PrEP uptake assessment							✓				
	Pill count							✓		✓		✓
	Self-report							✓		✓		✓
	Dried blood spot assessment									✓		

^a^PrEP: preexposure prophylaxis.

**Table 5:** Schedule of evaluations for study 2.

					Screening			Enrollment			Month 1			Month 2
**Eligibility assessment**														
	Pregnancy status			✓										
	HIV status			✓										
	Additional eligibility criteria			✓										
**Participant-reported assessments**														
	**Questionnaires**													
		Demographic information				✓								
		Intervention assessment				✓						✓		
		Decisional conflict				✓								
		Partner information				✓			✓			✓		
		Social harms				✓			✓			✓		
		Qualitative interviews							✓					

### Data Collection

Interviewer-administered questionnaires will be completed at the month 1 and final follow-up visits (month 3 for study 1 and month 2 for study 2). Questionnaires will assess study outcomes and associated social and behavioral measures to contextualize understanding of primary study outcomes, as summarized in Tables 4 and 5.

At each follow-up visit, pregnant women who have chosen oral PrEP will undergo adherence assessment through self-report and pill counts. In study 1, we will also collect dried blood spots at month 2 to assess adherence among all oral PrEP users.

Qualitative IDIs will be conducted at designated visits to assess women’s satisfaction with study counseling, session quality, and decision-making experience. Follow-up interviews will explore their experiences using the selected HIV prevention method and the perceived impact of study counseling. Additional feedback on the intervention and study procedures will be gathered.

IDIs with male partners and study staff will provide information on their experiences, perceptions of integrating SDM into routine care, and suggestions for improvements. A trained research assistant fluent in Chichewa and English will conduct the interviews using semistructured guides (Multimedia Appendix 1). Interviews will be audio recorded, transcribed, and translated into English for analysis.

### Retention

Once participants are enrolled, the study team will implement strategies to ensure high retention and minimize bias from loss to follow-up. Retention rates will be closely monitored, and challenges will be addressed proactively. Key strategies include providing a thorough explanation of visit schedules and procedures during informed consent, collecting detailed locator information at enrollment, and using timely reminders such as phone calls or SMS text messages (with participant consent). The study team will also follow up on missed visits through home or alternative off-site visits when feasible. Trained outreach workers will also conduct in-person follow-ups at participants’ homes or other agreed upon locations to enhance retention. If participants choose to discontinue their involvement in the study, we will document their stated reasons.

### Safety Monitoring

At each study visit, study staff will evaluate participants for social harms and adverse events. A social harm will be defined as a nonmedical untoward consequence of study participation, including difficulties in personal relationships, stigma, or discrimination from family or the community. An adverse event will be defined as any untoward medical occurrence in a study participant, including any unusual clinical features (eg, non–within-reference findings on physical examination or laboratory tests), symptom, or disease temporally associated with the individual’s participation in the study regardless of whether it is considered related to participation in the study.

### General Statistical Approach

Initial analyses will include descriptive statistics of the randomized study populations’ general characteristics (eg, sociodemographics) and each outcome. Any key characteristics that differ significantly by study arm will be treated as covariates in sensitivity analyses; otherwise, primary and secondary analyses will be unadjusted. The analysis will focus on estimation rather than hypothesis testing, with an α of .05 used throughout to compute 95% CIs. Exact statistical methods to estimate 95% CIs will be used in cases of small cell counts or analysis of continuous measures where n<30. Given potential for precision loss with exact CI methods and the pilot nature of these studies, we will use large-sample methods (eg, Wald CIs) when the nominal CI coverage level is tenable. Missing data are expected to be uncommon (≤10%), and a complete case analysis including only evaluable measures will be conducted by default. If missing data exceed 10%, multiple imputation methods may be used where relevant.

### Primary End Points

#### Overview

Primary study end points evaluated in both studies include intervention acceptability and appropriateness assessed via survey measures. The additional primary end point of intervention feasibility will be evaluated in study 1. Descriptive statistics will be calculated for primary outcomes among intervention arm participants. For each 4-item scale, item scores will be averaged to produce a composite score for each participant (range 1-5). Higher scores will indicate a greater level of acceptability, appropriateness, or feasibility. We will estimate the mean differences between arms for each primary outcome with a corresponding 95% CI and *P* value.

#### Intervention Acceptability (Studies 1 and 2)

We will evaluate intervention acceptability, defined as the extent to which participants perceive the intervention to be agreeable, palatable, or satisfactory. Acceptability will be assessed through participant self-report using a validated 4-item scale. This scale measures how agreeable and satisfactory women find the intervention (responses rated on a 5-point Likert scale ranging from “completely disagree” to “completely agree”). This assessment will be conducted at both month 0 and month 2 using the Acceptability of Intervention Measure scale [22].

#### Intervention Appropriateness (Studies 1 and 2)

We will also evaluate participant perceptions of intervention appropriateness, defined as the perceived relevance and usefulness of the intervention to support decision-making about HIV prevention methods. Appropriateness will be assessed through self-report using a validated 4-item scale to measure women’s perceptions of intervention relevance and usefulness (responses rated on a 5-point Likert scale ranging from “completely disagree” to “completely agree”) [22].

#### Intervention Feasibility (Study 1)

We will also evaluate participant perceptions of intervention feasibility, defined as the extent to which the intervention is deemed feasible or practical from their perspective. This feasibility of intervention measure will be administered at both month 0 and month 2. The items on the scale will be measured using a 5-point Likert scale, and individual responses will be averaged to obtain a score for each measure. Higher scores will indicate a greater level of feasibility [22].

### Secondary End Points

#### Decisional Conflict (Study 1)

The Decisional Conflict Scale (DCS) will be used to gauge women’s perceptions of decision uncertainty, satisfaction, clarity of personal values, and support for decision-making. This 16-item measure assesses these aspects on a 5-point scale, with scores totaling 100 points (mean rating multiplied by 25) [19,22-24]. A low DCS score will be identified using a cutoff of 25 out of 100, which has been linked to reduced decisional regret and enhanced choice retention. DCS scores will be computed based on women’s responses to statements, where higher scores indicate greater decisional conflict whereas lower scores indicate diminished conflict and heightened certainty in decision-making. The scale will be administered at the enrollment visit following completion of study counseling. Summary scores will be presented descriptively (mean and SD across participants in each arm by study) using an intention-to-treat approach. We will first compare group means of DCS scores by study arm and then examine the intervention’s effect on this outcome using a 2-sample Welch *t* test. If demographic variables related to decisional conflict differ substantively by study arm, we will instead use augmented inverse probability weights to conduct doubly robust estimation of the average intervention effect.

#### Study Procedure Feasibility (Study 2)

The feasibility of the study procedures refers to how effectively these procedures can be implemented in the study setting. To assess this feasibility, we will analyze study records and focus on several specific outcomes. These outcomes include the mean number of women screened weekly; the proportion of eligible women who enroll in the study; and retention rates, which encompass the proportion of women returning for each scheduled follow-up visit. In addition, we will measure the number of days required to meet both enrollment and retention targets, starting from initiation of enrollment and from the first scheduled follow-up visit, respectively. Descriptive statistics such as mean, SD, or percentages will be used to evaluate each of these feasibility outcomes.

### Exploratory End Points

#### PrEP Uptake (Studies 1 and 2)

We will assess PrEP uptake (ie, receipt of PrEP prescription) among participants who choose PrEP during the counseling session in both arms. This will be assessed through clinic or pharmacy records.

#### Retention With Functional Adherence (Study 1)

The clinical exploratory end point to be measured among participants using oral PrEP is retention in care with functional adherence to PrEP at 2 months. We will assess adherence to oral PrEP among participants taking it through tenofovir diphosphate (TFVdp) concentrations in participant dried blood spot samples. TFVdp will be measured using established liquid chromatography tandem mass spectrometry methods [25]. Using published thresholds [26], TFVdp concentrations will be categorized as shown in Table 6. Emtricitabine triphosphate concentrations will also be quantified and may be interpreted in an exploratory manner (eg, in the event that TFVdp is below the level of quantification). Participants will be considered for this composite outcome if they have any record of oral PrEP initiation following study-assisted referral. Participants retained at 2 months who meet the relevant definition of functional adherence (Table 6) will be categorized as retained with functional adherence.

**Table 6:** Adherence interpretation of tenofovir diphosphate (TFVdp) concentration levels.

Interpretation	Dried blood spot TFVdp concentration per punch (fmol)	
	Pregnant participants	Postpartum participants^a^
Approximately 7 doses per week	≥650	≥1050
2-6 doses per week	200-649	300-1049
<2 doses per week	<200	<300

^a^Participants will be enrolled during pregnancy but may deliver before the month 2 visit, when this outcome is assessed.

A treatment effect on PrEP adherence among participants who adopt PrEP will be explored as sample size and rates of adoption permit. We will compare the proportion of the sample retained in care with functional PrEP adherence at the 2-month follow-up between the 2 randomization arms using a linear binomial model. Women who are not retained at the 2-month follow-up will be counted as failures and will contribute to the analysis denominator. From the linear binomial model, the site-adjusted risk difference of being retained and in care with functional PrEP adherence for the intervention arm versus the control arm will be calculated, along with the corresponding 95% CI. In addition to the primary analysis, assessing functional PrEP adherence as a binary outcome, secondary analyses will compare the adherence categories between the study arms using a Wilcoxon rank sum test. This analysis will be restricted to women with an evaluable adherence score.

#### Intervention Fidelity (Studies 1 and 2)

With participant consent, intervention sessions will be digitally audio recorded. A randomly selected subset of sessions (approximately 20%) will be reviewed by a study team member using a structured fidelity assessment tool to evaluate intervention fidelity. Study staff will use recordings to evaluate whether counseling was delivered as expected (adherence to intended components and quality of counseling delivered) using the structured tool. Raters will score each counseling section as well as providing an overall score, with elements rated on a 5-point scale (0=“no effort”; 4=“exemplary effort”).

#### Qualitative Assessment of Intervention and Study Procedure Perceptions (Studies 1 and 2)

Thematic analysis will be used for the qualitative data [27]. The interviews will be transcribed and translated into English. Analysis will follow several steps. The first step is reading for content. We will read the data until content becomes intimately familiar. As the data are reviewed, emergent themes will be noted. The second step is coding. A list of codes will be created and documented in a codebook based on identified themes in addition to structural codes corresponding to initial interview questions. Two coders will independently code each transcript. To ensure intercoder reliability, 10% of the data will be double coded. The third step is data reduction. We will review the data related to each code to identify principal subthemes that reflect finer distinctions in the data. This entails taking an inventory of what is related to a given code, observing the variation or richness of each theme, and noting differences between individuals or among subgroups. The fourth step is data display and comparison. Matrices that categorize and display data will be used to help facilitate comparisons across the sampling groups. Throughout the analysis, the research team will engage in ongoing reflexivity through memos, considering how our positionalities, assumptions, and disciplinary backgrounds shape data interpretation. We will address key criteria for trustworthiness in qualitative research. Credibility will be promoted through iterative coding and team discussions to refine data summary and interpretation. Dependability will be established by documenting coding and other analytic decisions. Confirmability will be supported through cross-checking of interpretations within the research team. Finally, transferability will be communicated through detailed narrative description of both the research context and participants, allowing readers to assess the applicability of the findings to other settings and populations.

### Ethical Considerations

The study protocol and participant consent forms were approved by the Malawi National Health Science Research Committee (23/10/4210 and 23/10/4211) and the University of North Carolina at Chapel Hill Institutional Review Board (23-3102 and 24-0511). The 2 studies were prospectively registered in ClinicalTrials.gov (NCT06394323 and NCT06397690). Written informed consent was obtained from all study participants before taking part. Consent forms are available in Chichewa and English. Participants receive transport reimbursement equivalent to US $10 in Malawi kwacha at the prevailing exchange rate in accordance with the local ethics committee’s recommendation. Trial NCT06394323 was registered on April 26, 2024. Trial NCT06397690 was registered on April 19, 2024.

All data are deidentified and stored securely on locked cabinets or encrypted servers, with transcripts anonymized. To ensure the security of the data and maintain participant confidentiality, all interviews and data collection will take place in private areas at the health facility. Any audio recordings made during qualitative interviews will be promptly transferred to a secure computer, and the original recordings will be erased. The transcripts of these recordings will not include any identifying information. Similarly, paper clinic charts and other documents will be deidentified to remove any personal details. Electronic files will be stored on encrypted devices with password protection to further ensure data security. These measures are in place to safeguard the privacy of the participants and maintain the integrity of the research data.

## Results

Data collection commenced on July 30, 2024, for study 1 and on February 10, 2025, for study 2. MyChoice study 1 has enrolled 100 participants, and MyChoice study 2 has enrolled 50 participants, with all participants completing the study follow-up. Data analysis is expected to be completed by November 2025.

## Discussion

Our feasibility pilot study seeks to assess the acceptability and appropriateness of the MyChoice intervention to facilitate informed choices about PrEP use during pregnancy. The results of this study will provide novel evidence on the feasibility of delivering PrEP SDM in an antenatal care context and the potential utility of this approach to help women make personally appropriate choices regarding HIV prevention methods during pregnancy and breastfeeding. Currently, there is limited understanding of effective strategies to engage pregnant women in PrEP and identify appropriate candidates within this population. While many pregnant women in sub-Saharan Africa meet the HIV risk criteria for PrEP, such as having a partner with known HIV status or a history of sexually transmitted infections [28,29], studies have shown that women meeting these criteria often exhibit low adherence to PrEP [6-8]. This low adherence may indicate that assessed HIV risk alone is not an adequate motivator for sustained PrEP use.

Fit and motivation for PrEP use are crucial for promoting adherence, and an SDM process may play a key role in achieving both [12]. Unlike ART, the choice to use PrEP is highly sensitive to individual preferences. While PrEP offers the advantage of being a woman-controlled HIV prevention method, it may not be the right choice for every woman during her pregnancy and while breastfeeding. Evidence of variation in women’s preferences for HIV prevention methods based on personal values indicates the need for decision support [30-33]. SDM can support user fit by promoting value-aligned decisions and reducing decisional conflict—defined as uncertainty about the best choice among competing options or discomfort with the decision made [34]. Our study provides an opportunity to assess SDM in the context of choosing between oral PrEP and CAB-LA for HIV prevention during the perinatal period. SDM helps reduce decisional conflict by improving individuals’ understanding of available options and their benefits and risks and clarifying personal values. Both theoretical frameworks and empirical evidence show that SDM promotes value-congruent decisions and supports adherence to treatments such as ART, as well as therapies for conditions such as depression [35,36]. In HIV care, SDM is highly accepted; strongly desired by patients; and linked to better ART adherence by promoting patient engagement, adherence self-efficacy, confidence in the treatment plan, and satisfaction with the patient-health care provider relationship [37-39]. We need to help women decide which HIV prevention method best fits their needs and preferences as this method will be the most likely to be used effectively [40].

Few studies have evaluated PrEP decision support interventions in women’s health settings. A pilot study of women in addiction treatment receiving a PrEP decision aid observed increased interest in PrEP from 25% to 89% and increased likelihood of a scheduling a health care provider visit [41]. A trial of a digital PrEP decision support tool developed for adolescent girls and young women in South Africa demonstrated higher PrEP persistence among young women receiving the tool [42]. With the MyChoice intervention, we aim to provide in-clinic PrEP decision support tailored to pregnant women, and we will evaluate the potential effect of the intervention on PrEP persistence and adherence in a future trial. To our knowledge, MyChoice is the first empirically evaluated PrEP decision support intervention for perinatal populations.

Limitations to the design of these pilot trials should be noted. As pilot studies, these trials are not designed to detect an intervention effect on the ultimate outcome of PrEP use but are rather designed to assess the appropriateness, acceptability, and feasibility of the study intervention. If the study intervention and design prove feasible, we will evaluate the effect of the intervention on PrEP use in a larger randomized controlled trial in the future. The self-reported primary and secondary end points in these studies are subject to social desirability bias. To mitigate this potential bias, we ensured that the study staff delivering the intervention were separate from those conducting study questionnaires and interviews with participants and also made clear to participants that there were no right or wrong answers to any questions and that we welcomed all feedback, both positive and negative.

PrEP presents an opportunity to dramatically reduce the elevated risk of HIV acquisition in pregnant and breastfeeding women in sub-Saharan Africa, and SDM may complement the potential impact of PrEP by identifying the appropriate users and promoting adherent use. No evidence-based approaches for PrEP SDM in pregnancy exist. Thus, we aim to fill this gap with these pilot studies to determine the feasibility, acceptability, and appropriateness of a novel SDM intervention and associated study procedures to inform a future efficacy trial.

## Appendix

Multimedia Appendix 1 Interview guide.

Multimedia Appendix 2 Peer review report from HIBI - HIV/AIDS Intra- and Inter-personal Determinants and Behavioral Interventions Study Section, AIDS and Related Research Integrated Review Group (National Institutes of Health, USA).

## Abbreviations

CAB-LA: long-acting cabotegravir
DCS: Decisional Conflict Scale
IDI: in-depth interview
PrEP: preexposure prophylaxis
SDM: shared decision-making
SOC: standard of care
TFVdp: tenofovir diphosphate
