# Supplementary material for: Supporting Oral and Long-Acting HIV Preexposure Prophylaxis Decision-Making Among Pregnant Women (MyChoice Intervention): Protocol for 2 Pilot Randomized Controlled Trials
Source: JMIR Res Protoc. 2025 Nov 13;14:e76442. doi: 10.2196/76442 (PMC12661230; doi:10.2196/76442)
Supplement: Multimedia Appendix 1 [file resprot_v14i1e76442_app1.docx]

**Interview Guide 1 : SDM participants interview 1 (of 2)**

*Thank you for taking the time to talk with me today. In this interview we will talk about your experience choosing an HIV prevention with the support of the study counselor, and your feelings about using that method since you started. There are no right or wrong answers to the questions I am going to ask. I would like to hear about your experience, opinions, and ideas from your own perspective. We want to hear all of your opinions about the tool, both positive and negative; negative feedback will help us improve how we support women to make this decision. You may skip any questions you do not want to answer, and you may end the interview at any time. As we go through the interview, please stop me at any time if you have any questions or comments.*

Do you have any questions before we begin?

**Opening grand tour questions**

1. How has your pregnancy been going so far?
   - What has your relationship with your partner been like during your pregnancy?
2. Can you tell me how did you decided to join the study?
   - What motivated you to join?
   - What was your understanding of your HIV risk when you joined the study?

**Decision-making experience**

1. Before you did the study counseling, what did you know about the available HIV prevention methods?
   - What did you know about PrEP? What experience did you have with it? (Probe: for the other kind of PrEP if they have only mentioned one type)
   - What did you know about condoms? What experience did you have with it? (Probe: for the other kind of condoms if they have only mentioned one type)
2. Can you describe the decision-making session you completed with the study counselor? What happened in the session?
   - Was your partner or anyone else present?
3. Can you describe your conversation with the counselor about the HIV risk factors that applied to you?
   - How helpful or not helpful was this part of the counseling? What are the reasons for that?
   - What was your understanding of your HIV risk after this discussion?
   - [*If partner present*] How did you feel about having your partner present for this discussion? What are the reasons for that?
4. Tell me about the values clarification exercise part of the counseling. This is when the counselor shared different cards with you and asked you to pick values that mattered to you.
   - What values did you pick and how did they guide your decision?
   - How did this exercise help you understand different HIV prevention methods?
   - How helpful or unhelpful was this section? What are the reasons for that?
5. What HIV prevention method(s) did you choose and why?
   - What factors were most influential in your decision?
   - Were there any specific benefits or risks that influenced your decision?
   - How did you weigh the options and make your choice?
   - What role did the counselor and your partner play in helping you make this decision?
   - What other factors influenced your decision?
6. How confident do you feel about the decision you made? What are the reasons for that?
   - Did you experience any internal conflicts or uncertainties during the decision-making process? What were the reasons for that?
   - How did the counseling/counselor influence how you felt about your decision?
   - How did your partner or others influence how you felt, if at all?
7. Looking back at when you made your decision, what were the most significant challenges you faced in making this decision?
   - Were there any specific factors or moments during the process that made the decision difficult?
   - How did you navigate through the challenges to reach your decision?
8. How informed do you think you were about the advantages and disadvantages of the option(s) that you chose? What are the reasons for that?
   - Were there any areas where you wish you had more information before making the decision?
   - Was there information you were given that was unclear?
9. Did you seek support or advice from others during the decision-making process on whether to use or not use PrEP?
   - How did the support you received influence your decision, if at all?
   - Were there any external perspectives that you found particularly helpful or influential?
   - Were there any conflicting opinions or advice that you had to consider?
10. To what extent do you feel you were able to make your choice freely on your own? What are the reasons for that?
    - Were there any instances where you felt pressured or influenced towards a particular HIV prevention method?
11. Looking back, would you make the same decision now as you did then? What are the reasons for that?

**Counseling Top of Form**

**Acceptability/Appropriateness:**

1. How satisfied are you with the decision-making counseling you received to choose an HIV prevention methods? What are the reasons for that?
   1. What aspects of the counseling contributed to your satisfaction?
   2. How did the counseling influence your decision?
2. Were there any aspects of the counseling you felt could be improved?
   1. Were there any parts that were unhelpful or unnecessary?
   2. Did you wish for additional information or support?
3. What parts of the counseling were *most* helpful? What are the reasons for that?
4. What parts of the counseling were *least* helpful? What are the reasons for that?
5. How comfortable did you feel discussing your concerns and uncertainties during the decision-making counseling sessions?
   1. Did the decision-making counseling intervention adequately consider your individual circumstances and preferences in guiding you towards an appropriate HIV prevention method?
6. [If Applicable] Can you share your experiences and thoughts on involving your partner or someone else other than the counselor in the decision-making process?
   1. How did you feel about the way your counselor engaged your partner in the counseling? What are the reasons for that?
   2. What could be improved about how your partner was included/engaged in the counseling?
   3. Would you involve your partner or this person in your decision-making processes in the future?
7. How did you feel about the duration of the decision-making counseling sessions? E.g., long, short, etc.
8. How did you feel about the person who conducted the counseling with you?

What did you like about the counselor?

What could they have done better?

**Counseling/Study procedure feasibility:**

*Now I would like to get your thoughts on other aspects of your engagement in the study and how they were conducted.*

1. How did you feel about the recruitment process for the study?
   - What did you like about the process?
   - What could have been better about the process?
2. Once you expressed interest in the study, a staff member asked you questions to see if you were eligible to participate (e.g., HIV status, pregnancy)? How did you feel about this process?
   - What could have been better about the process?
3. Before you completed the counseling, a study staff member gave you information about the study and asked you to sign to indicate your consent to participate. How did you feel about this process?
   - To what extent did you understand what you were consenting to?
   - What could have been better about the process?

4. Is there any other feedback you would like to share about your experience in the study so far?

**Interview Guide 2 : SDM participants interview 2 (of 2)**

*Thank you for taking the time to talk with me today. In this interview we will talk about your experience with the study since we last talked and your experiences using the HIV prevention method you selected. There are no right or wrong answers to the questions I am going to ask. I would like to hear about your experience, opinions, and ideas from your own perspective. We want to hear all of your opinions and experiences, both positive and negative; negative feedback will help us improve how we support women to make this decision. You may skip any questions you do not want to answer, and you may end the interview at any time. As we go through the interview, please stop me at any time if you have any questions or comments.*

Do you have any questions before we begin?

**Experience with selected HIV prevention method:**

1. How has your experience been using [PrEP, condoms] so far?
2. How have your feelings changed about using [PrEP, condoms] since you first made the decision?
   - How confident do you feel in your decision to continue using [PrEP, condoms]?
   - Can you tell me more about any doubts or uncertainties you've experienced?
3. What challenges, if any, have you faced in using [PrEP, condoms]?
   - How has your partner helped or hindered your ability to use [PrEP, condoms]?
   - How have other people in your life affected your use of [PrEP, condoms]?
   - Were there any times you wanted to stop using [PrEP, condoms]? What were the reasons for that?
4. What strategies or support, if any, have helped you use [PrEP, condoms] consistently?
5. [PrEP users only] Have you shared your decision to use PrEP with your partner?

[If yes] Can you tell me about that conversation?

- - How did he react when you told him?
  - How does he feel about it now?
  - What role does he play in your use of PrEP?

[If no] What are the reasons you decide to not tell him?

1. Is there anything you wish you had known or considered at the time you made the decision to use [PrEP, condoms]?
2. If you had the decision to make over again, what choice would you make? What are the reasons for that?

**[PrEP users only] Change in initial values and current use:**

1. Thinking back to when you first decided to use PrEP, *what motivated you* to use it? How has this changed, if at all?

- How do these motivations affect your use of PrEP?

1. Thinking back to when you first decided to use PrEP, what were you *most concerned about*? How has this changed, if at all?

- How have these concerns affected your use of PrEP?

1. Thinking back to when you first decided to use PrEP, how confident were you about your choice? How has this changed, if at all?

- How has this impacted your use of PrEP, if at all?

**Experience with Study Assessments and Procedures:**

*Thank you for sharing your thoughts about the HIV prevention method you chose. Now I’d like to ask about your experience participating in this study.*

1. Please tell me about your experience with the study surveys that you completed. Remember that at each visit you have completed a questionnaire with shorter questions than these in-depth interview with me.
   - How do you feel about the questions asked in these surveys?
   - How do you feel about the length of these survey?
   - What could be improved about these surveys?
2. How do you feel about these interviews that you and I have done together?
   - How do you feel about the questions asked in these interviews?
   - How do you feel about the length of these interviews?
   - What could be improved about these interviews?
3. How do you feel about the frequency and timing of the study visits you’ve completed?
   - How easy or hard is it to come for these visits? What are the reasons for that?
   - How easy or hard is it to stay for the duration of these visits? What are the reasons for that?

**[PrEP users only] Experience with adherence assessments:**

1. At each visit we have asked you to bring your PrEP pill bottle with you so we can count the pills. How do you feel about this?
   1. Do you understand the purpose is?
   2. How comfortable do you feel with having this done?
   3. What could be improved about the way we do this?
2. You will remember that at the last visit took a small sample of your blood to test the amount of PrEP in it. How did you feel about this?
   1. Do you understand the purpose is?
   2. How comfortable do you feel with having this done?
   3. What could be improved about the way we do this?

**Overall Study Experience and Suggestions for Improvement:**

1. How would you describe your overall experience as a participant in this study?
   - What aspects have you found *most* engaging or valuable? What are the reasons for that?
   - What aspects have you found *least* engaging or valuable? What are the reasons for that?
2. Would you recommend this study to other women? What are the reasons for that?
3. Would you participate in a similar study again in the future if it were relevant to you? What are the reasons for that?

**Interview Guide 3 :Male Partner IDI Guide**

Thank you for taking the time to talk with me today. In this interview we will talk about your perspective of your wife/partner’s experience choosing an HIV prevention with the support of the study counselor, and your role in this decision. There are no right or wrong answers to the questions I am going to ask. I would like to hear about your experience, opinions, and ideas from your own perspective. We want to hear all of your opinions about your experience, both positive and negative; negative feedback will help us improve how we support couples to make this decision. You may skip any questions you do not want to answer, and you may end the interview at any time. As we go through the interview, please stop me at any time if you have any questions or comments.

Do you have any questions before we begin?

**General**

1. Could you tell me about your relationship with the mother of your child?
2. How did you first learn about the decision-making study that your partner is taking part in?
   - What did you think when you first heard about it?

**Involvement in Decision Making:**

1. Can you tell me about the conversation you had with your partner about taking part in the decision-making counseling?
   - What were your initial thoughts and reactions when your partner approached you about this?
   - What led you to agree to take part in the counseling?
   - What concerns, if any, did you have going into the counseling?
2. What was your knowledge of the HIV prevention methods discussed before you completed the counseling session?
   - What did you know about PrEP? What experience did you have with it?
   - What did you know about condoms? What experience did you have with it?
3. How were you involved in the decision-making counseling with your partner?
   - Can you describe the session?
   - What did you understand your role to be in the counseling session?
   - How did the counselor involve you in the session?
   - In what ways did your partner seek your opinion during or outside of the session?
4. How did you feel about your involvement in the decision-making process for your partner's HIV prevention?
   - What did you like most about the session? What are the reasons for that?
   - Were there any concerns you had during the conversation?
   - Reflecting back, is there anything you would have preferred to be different about the way you were included in these decisions regarding HIV prevention?
   - What information was new to you?
   - Was there anything you learned that surprised you? Please explain.
5. Can you describe your role in the part of the counseling on the HIV risk factors that applied to your partner?
   - How did you feel being present for this discussion? What are the reasons for that?
   - How helpful or not helpful was this part of the counseling? What are the reasons for that?
   - What was your understanding of her HIV risk after this discussion?
   - What was your understanding of your HIV risk after this discussion?
6. How do you think your involvement affected your partner’s choice of prevention method?
   - How did you see your role in the overall process? Were you more of a facilitator, contributor, or observer during the session?
   - Were there any particular aspects where your input had a significant impact on the decisions that were made?
7. Please tell me about any discussions you had with your partner after the counseling session.
   - Did you or your partner change your opinion on the best choice after the counseling? What are the reasons for that?

**Satisfaction in the Decision-Making Session:**

1. Looking back on the decision-making session, how satisfied are you with the outcome of the decision-making session? What were the reasons for that?
   - What specific factors contribute to your level of satisfaction or dissatisfaction with the outcomes?
2. How confident did you feel about the right choice for your partner?
   - What, if anything, were you uncertain about?
   - What would have helped address this uncertainty?
   - Was there anything you or your partner did to seek clarity?
3. Did you feel your opinions were valued during the session? What are the reasons for that?
   - Can you recall any specific instances where your concerns were addressed or integrated into the discussions?
4. Were there any aspects of the decision-making process that could have been improved?
   - What additional information, if any, would you have wanted?
   - What else would you have wanted to talk about with the counselor if anything?
   - How could the session have been made more comfortable or convenient for you?

**Experience Supporting Partner's PrEP Use (if applicable):**

1. How do you feel about your partner using PrEP?

- What do you like about it?
- What do you dislike about it?
- Is there anything about your partner using PrEP that you did not expect?

1. Are there any ways that you help your partner take PrEP? Please tell me about that?

- Are there any ways that you motivate her to keep taking PrEP?
- Are there any ways that you help remind her to take PrEP?
- How did you develop these strategies?

1. What challenges, if any, have you encountered while supporting your partner's PrEP use?

- How regularly does she take PrEP? What are the reasons for that?
- What challenges has she faced to take PrEP? How have you helped her overcome these challenges?

1. How has supporting your partner's PrEP use impacted your relationship, if at all?

**[For HIV+ partners only] Impact on Motivation to Adhere to ART (if applicable):**

1. Have you ever tested for HIV? What were the results?
2. [If ever tested positive] Are you currently taking ART?

- How long have you been taking ART?

1. [For HIV+ partners] How, if at all, has your experience with the study affected your feelings about living with HIV?
2. [For ART-using partners partners] How, if at all, has your experience with the study affected your use of ART?
3. [For ART-using partners of PrEP users] Since your partner has been taking PrEP, how has your use of antiretroviral therapy (ART) changed, if at all? What are the reasons for that?

- Do you feel more or less motivated to take your ART since she started using PrEP? What are the reasons for that?
