# Supplementary material for: Supporting Oral and Long-Acting HIV Preexposure Prophylaxis Decision-Making Among Pregnant Women (MyChoice Intervention): Protocol for 2 Pilot Randomized Controlled Trials
Source: JMIR Res Protoc. 2025 Nov 13;14:e76442. doi: 10.2196/76442 (PMC12661230; doi:10.2196/76442)
Supplement: Multimedia Appendix 2 [file resprot_v14i1e76442_app2.pdf]

Telephone: + 265 789 400
Facsimile: + 265 789 431

All Communications should be addressed to: The Secretary for Health

In reply please quote No. MED/4/36c
Ministry of Health
P.O. Box 30377
Lilongwe 3
Malawi

Ref. No. MED/4/36c **27th October, 2023**

Lauren Hill
UNC Project

Dear Sir/Madam,

Protocol # 23/10/4211: Supporting Pre-Exposure Prophylaxis Decision Making Among Pregnant Women: A Pilot Study.

Thank you for the above titled communication that you submitted to the National Health Sciences Research Committee (NHSRC) for review.

The committee **reviewed** and **did not approve** the above named study until you **resubmit for full committee** after addressing the following concerns:

1. The study title should indicate the place of study
2. The study objectives should be SMART
3. There is no facility stamp on the signing sheet in the NHSRC form 101.
4. Elaborate where there is conflict of interest in the summary.
5. In the study design the researcher has indicated that their two nested studies, the committee suggests that they should be submitted separately.
6. The appendices are not appearing in the protocol
7. The Gant chart has no time indication work plan.
8. Resubmit a clean document
9. Indicate in the study that you are taking the drug (CABLA) from the national pilot.

Kind regards from the NHSRC Secretariat.

....

For: **CHAIRPERSON, NATIONAL HEALTH SCIENCES RESEARCH COMMITTEE**
Promoting Ethical Conduct of Research[1]

Ministry of Health
27 OCT 2023
Research Department
P.O. Box 30377, Lilongwe

Executive Committee: *Dr. M. Joshua (Chairperson), Dr. F. Sinyiza (Vice-Chairperson)*
Registered with the USA Office for Human Research Protections (OHRP) as an International IRB
IRB Number IRB00003905 FWA00005976
